# Comparative effectiveness of combination therapy with SGLT‐2 inhibitors and GLP‐1 RAs compared with SGLT‐2 inhibitors in individuals with type 2 diabetes: A prevalent new‐user cohort study

**DOI:** 10.1111/dom.70523

**Published:** 2026-02-22

**Authors:** Gregor A. Maier, Beata Hennig, Wolfgang Rathmann, Oliver Kuss

**Affiliations:** ^1^ Institute for Biometrics and Epidemiology, German Diabetes Center Leibniz Center for Diabetes Research at Heinrich Heine University Düsseldorf Germany; ^2^ Department of Medicine and Health Services Research BARMER Health Insurance Wuppertal Germany; ^3^ German Center for Diabetes Research München‐Neuherberg Germany; ^4^ Centre for Health and Society Medical Faculty and University Hospital Düsseldorf, Heinrich Heine University Düsseldorf Germany

**Keywords:** GLP‐1 analogue, observational study, pharmacoepidemiology, real‐world evidence, SGLT2 inhibitor, type 2 diabetes

## Abstract

**Aims:**

To evaluate the effectiveness of combination therapy with sodium‐glucose cotransporter 2 (SGLT‐2) inhibitors and glucagon‐like peptide‐1 receptor agonists (GLP‐1 RAs) compared with continued SGLT‐2 inhibitor therapy in routine practice among individuals with type 2 diabetes.

**Materials and methods:**

We used nationwide BARMER health claims data and implemented a prevalent new‐user design. Individuals initiating a GLP‐1 RA, either simultaneously with SGLT‐2 inhibitors or during ongoing SGLT‐2 inhibitor exposure, were matched to SGLT‐2 inhibitor continuers using hybrid exposure sets and time‐conditional propensity scores. The study cohort included individuals enrolled between 2013 and 2023, with possible follow‐up including 2024. The primary outcome was all‐cause mortality. Secondary outcomes included a modified cardiovascular composite (all‐cause mortality, myocardial infarction, stroke), heart failure, nephropathy, and renal failure. Cox proportional hazards models estimated hazard ratios (HRs) and 95% confidence intervals (CIs).

**Results:**

Among 21 664 matched pairs with a median follow‐up of 1.3 years, combination therapy was associated with a 29% lower hazard of all‐cause mortality (HR 0.71, 95% CI 0.63–0.80), consistent across subgroups and sensitivity analyses. Hazard reductions were also observed for the modified cardiovascular composite (HR 0.81, 95% CI 0.74–0.88) and heart failure (HR 0.78, 95% CI 0.68–0.89).

**Conclusions:**

In this large real‐world cohort, combination therapy with SGLT‐2 inhibitors and GLP‐1 RAs was associated with a lower hazard of all‐cause mortality, while most secondary cardiorenal outcomes showed generally favourable but imprecise estimates. These findings suggest that sustained concurrent use of both drug classes may offer meaningful clinical benefits in routine practice, although residual confounding cannot be fully excluded.

## 1 INTRODUCTION

Sodium‐glucose cotransporter 2 (SGLT‐2) inhibitors and glucagon‐like peptide‐1 receptor agonists (GLP‐1 RA) have transformed type 2 diabetes management due to their well‐established cardiovascular and renal benefits.^1^, ^2^ SGLT‐2 inhibitors primarily offer cardioprotection through hemodynamic and renovascular effects,^3^ while GLP‐1 RAs work via anti‐inflammatory pathways,^4^ central nervous system modulation,^5^ and delayed gastric emptying.^6^ Given these complementary mechanisms, there has been increasing interest in combining both drug classes. While both drug classes individually have strong recommendations for individuals with cardiovascular risk factors, current guidance on combining these mechanistically complementary agents remains cautious and reflects limited evidence.^2^

Despite demonstrated benefits in efficacy parameters like HbA1c reduction or weight loss,^7^ real‐world data on combination therapy with SGLT‐2 inhibitors and GLP‐1 RAs—compared to either drug class alone—remains scarce. Most studies would limit their analysis to incident new‐users of both drug classes (individuals initiating both drugs simultaneously without prior use of either drug class). This approach excludes prevalent new‐users (individuals who initiate one drug while already taking the other), such as those who add GLP‐1 RA to an existing treatment with SGLT‐2 inhibitors, thereby limiting the generalizability of results.

While cardiorenal benefits from combining both drug classes have been reported,^8^ only one study to date has employed a prevalent new‐user design to emulate a randomized controlled trial (RCT).^9^ This study using UK data reported consistent benefits for combination therapy compared to either drug class alone, appropriately reflecting routine clinical practice where individuals initiate either drug sequentially—an approach acknowledged in the ADA's Standards of Care.^2^

Nevertheless, evidence remains limited to this single study,^9^ highlighting the need for further research across diverse healthcare systems. Therefore, we aimed to investigate combination therapy with SGLT‐2 inhibitors and GLP‐1 RAs compared with continuing SGLT‐2 inhibitors on the incidence of all‐cause mortality and cardiorenal outcomes in the first nationwide study utilizing a prevalent new‐user design.

## 2 MATERIALS AND METHODS

### 2.1 Causal estimand

The targeted causal estimand was the effectiveness of combined incident new‐use (simultaneous initiation of SGLT‐2 inhibitors and GLP‐1 RAs) and prevalent new‐use (initiating a GLP‐1 RA while continuously exposed to SGLT‐2 inhibitors). Thus, we addressed the causal question: *Among individuals in the matched study cohort, what would have been if initiators of combination therapy who remained on combination therapy had initiated and remained on SGLT‐2 inhibitor therapy or had continued and remained on SGLT‐2 inhibitor therapy?* ^10^, ^11^

### 2.2 Sources of data

We used nationwide health claims data from one of Germany's largest statutory health insurance funds (BARMER, Wuppertal, Germany). The BARMER scientific data warehouse contains longitudinal individual‐level data on approximately 9 million insured individuals and has been used in prior pharmacoepidemiological research.^12^ The database includes a broad range of routinely collected data such as information on sociodemographic characteristics, health insurance enrolment status, inpatient and outpatient medical encounters coded according to ICD‐10 in its German modification, surgical and procedural codes (Operationen‐ und Prozedurenschlüssel [OPS]), as well as dispensed medications coded according to the German version of the international Anatomical Therapeutic Chemical (ATC) classification system.

### 2.3 Study design

We implemented a prevalent new‐user design.^13^ First, we established a base cohort that comprised individuals who were incident new‐users of SGLT‐2 inhibitors (dapagliflozin, empagliflozin, ertugliflozin, or canagliflozin) between 1 January 2013 and 31 December 2023. The base cohort index date corresponded to the date of an individual's first SGLT‐2 inhibitor dispensing. Therefore, we excluded individuals who were only exposed to GLP‐1 RAs, had an unclear health insurance status, had less than 1 year of insurance coverage before the base cohort index date, had no ambulatory encounter during that year, did not meet the criteria for verifiable type 2 diabetes (definitions provided in Table S1, Supporting Information), had missing information on sex, were younger than 18 years, or received multiple active ingredients or multiple dispensings of SGLT‐2 inhibitors on the base cohort index date.

Next, we formed the study cohort by following these individuals and collecting subsequent dispensings of SGLT‐2 inhibitors within continuous therapy. We defined continuous therapy using an on‐treatment approach with a 180‐day refill interval considering therapy as discontinued if no refill occurred within this interval. Rather than one observation per individual, each dispensing was treated as a separate row, meaning that one individual could contribute multiple observations over time. The unit of analysis therefore shifted from the individual to each of that individual's dispensings.

We then identified individuals who initiated a GLP‐1 RA. This GLP‐1 RA dispensing was classified as either incident new‐use of combination therapy if it occurred on the same date as the first SGLT‐2 inhibitor dispensing or prevalent new‐use if it occurred during continuous SGLT‐2 inhibitor therapy.

To ensure comparability, dispensings—whether GLP‐1 RA or SGLT‐2 inhibitor—were excluded if no ambulatory encounter was recorded in the preceding year or if multiple dispensings or active ingredients occurred on the same date.

For each combination therapy user, a hybrid exposure set was created to identify eligible reference dispensings from individuals who continued SGLT‐2 inhibitor therapy that matched the combination therapy user's treatment history at that specific timepoint. This ensures that individuals are compared at an equivalent stage of their disease progression and treatment history. A detailed definition of hybrid exposure sets and possible matching timepoints is provided in Figure S1.

### 2.4 Potential confounders

We adjusted for a total of 64 covariates to account for potential confounding. All covariates were measured prior to or on the date of the drug dispensing that defined (for GLP‐1 RA initiators) or corresponded to (for reference dispensings) membership in the respective hybrid exposure set. These covariates included sociodemographic characteristics, diabetes duration and diabetes‐related complications, comorbidities, procedures, co‐medications, glucose‐lowering therapies, markers of health‐seeking behaviour, healthcare utilization, and cohort entry year. Detailed definitions and assessment windows are provided in Table S2.

### 2.5 Time‐conditional propensity scores

We calculated a time‐conditional propensity score (TCPS) for initiating GLP‐1 RA (becoming a combination therapy user) versus continuing SGLT‐2 inhibitors using multivariable conditional logistic regression with 64 pre‐specified covariates, conditional on the hybrid exposure sets. Unlike conventional propensity scores, which are estimated once per individual, the TCPS was recalculated for each dispensing using the updated covariates from the timepoint of inclusion in the hybrid exposure set. By updating the propensity score for each hybrid exposure set, this approach provides a time‐conditional measure of the propensity of initiating GLP‐1 RA versus continuing SGLT‐2 inhibitors, accounting for temporal changes in covariates.

We ensured positivity by trimming hybrid exposure sets where the combination therapy users' TCPS fell outside the TCPS range of the eligible reference dispensings. One‐to‐one matching without replacement was conducted in chronological order, starting with the earliest combination therapy user by calendar time,^13^ based on both the hybrid exposure set (to ensure identical treatment history) and the nearest TCPS (to balance all measured covariates). Additionally, we used a calliper width of 0.2 times the standard deviation of the linear predictor. Once a reference dispensing was matched, it was no longer eligible for subsequent matching.

### 2.6 Combination therapy

Combination therapy was defined as the period of concurrent use of both SGLT‐2 inhibitors and GLP‐1 RAs. Consistent with our definition for SGLT‐2 inhibitors, a 180‐day refill interval was also applied to GLP‐1 RAs to determine continuous use. This period ended when coverage for either drug lapsed.

### 2.7 Primary and secondary outcomes

The primary outcome was all‐cause mortality. Secondary outcomes included a modified cardiovascular composite (all‐cause mortality, myocardial infarction, or stroke), its individual components, heart failure, diabetic nephropathy, and renal failure. All outcomes except all‐cause mortality were defined by ICD‐10‐GM hospital diagnosis codes in primary position capturing both incident and recurrent hospitalizations. For detailed outcome definitions, see Table S3.

### 2.8 Follow‐up

Accumulation of follow‐up time started on the study cohort index date (date of first GLP‐1 RA dispensing or date of matched SGLT‐2 inhibitor dispensing for reference individuals) and continued until the outcome, death (unless death was the primary outcome in analyses of all‐cause mortality or the modified cardiovascular composite outcome), end of health plan enrolment, treatment discontinuation, initiation of GLP‐1 RA (for reference individuals), or the end of the study (31 December 2024).

References censored due to GLP‐1 RA initiation could re‐enter as combination therapy users if a match was available, with follow‐up starting 1 day after GLP‐1 RA initiation. No other re‐entries were permitted, and only the first drug exposure episode was used.

### 2.9 Subgroup and sensitivity analyses

We assessed potential effect measure modification by including interaction terms between treatment group (combination therapy versus continuing SGLT‐2 inhibitors) and sex, as well as cardiovascular disease (CVD).

In addition, we conducted 10 sensitivity analyses to evaluate the robustness of the result for our primary outcome. First, we assessed whether the effect varied with different matching procedures: one‐to‐one nearest neighbour matching without a calliper, matching the latest GLP‐1 RA initiator first, and one‐to‐two nearest neighbour matching. In addition, we investigated whether including individuals who were initially matched as SGLT‐2 inhibitor continuers and later re‐entered the cohort as combination therapy users influenced the results. For this analysis, re‐entry into the cohort was not allowed. To address potential bias due to informative censoring from treatment discontinuation, initiation of GLP‐1 RA among reference individuals, or administrative censoring (end of health plan enrolment or end of study), we applied stabilized time‐varying inverse probability of censoring weighting (IPCW).^14^ To evaluate the impact of potential exposure misclassification, we repeated the analysis using a 120‐day and 90‐day refill interval and an intention‐to‐treat (ITT) exposure definition. Finally, we constructed prescription‐based and time‐based exposure sets as originally described.^13^ In these analyses, exposure sets were defined solely by the number of prior SGLT‐2 inhibitor dispensings or time since base cohort index date, relaxing the stricter hybrid exposure set criteria.

### 2.10 Statistical analysis

This observational study was conducted and reported in accordance with the STROBE guidelines for observational research.^15^ Characteristics of individuals were summarized using counts and proportions for categorical variables and means with standard deviations for continuous variables. Covariate balance was evaluated using absolute standardized differences (ASDs), with values ≥10% considered indicative of meaningful imbalance.^16^ Event rates per 1000 person‐years were calculated based on the Poisson distribution with corresponding 95% confidence intervals. For the comparative analyses, Cox proportional hazards models were used to estimate hazard ratios and 95% confidence intervals. Individuals initially matched as a reference could potentially contribute two observation periods, the first period as a reference continuing SGLT‐2 inhibitors, and the second period as a combination therapy user upon GLP‐1 RA initiation. To account for this within‐individual correlation of person‐time we calculated robust (sandwich) standard errors.

Absolute risks were derived using Kaplan–Meier methods. Risk differences (RDs) were estimated for up to 5 years of follow‐up, with 95% confidence intervals obtained via bootstrap resampling with 1000 iterations. All statistical analyses were performed using R (R Foundation for Statistical Computing, Vienna, Austria, http://www.R-project.org/).

## 3 RESULTS

**FIGURE 1:**
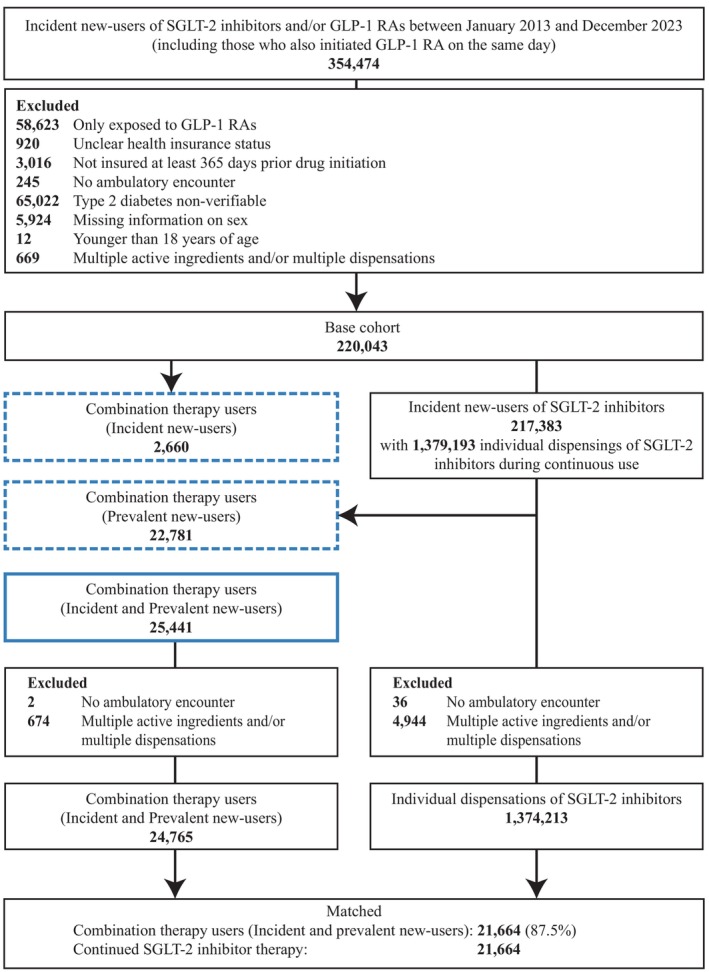
Flowchart illustrating study cohort selection. The diagram shows eligibility criteria, the number of excluded individuals, and the individuals used to derive the base and study cohorts, including assignment to combination therapy with SGLT‐2 inhibitors and GLP‐1 RAs versus continued SGLT‐2 inhibitor therapy. The upper blue box with a dashed line indicates incident new‐users of combination therapy (individuals who simultaneously initiated SGLT‐2 inhibitors and GLP‐1 RAs), while the lower blue box with a dashed line indicates prevalent new‐users (individuals who initiated GLP‐1 RA during continued SGLT‐2 inhibitor therapy). The lower blue box with a solid line represents both incident and prevalent new‐users. Figure adapted from Simms‐Williams et al.^9^ GLP‐1 RA, glucagon‐like peptide‐1 receptor agonists; SGLT‐2, sodium–glucose cotransporter 2.

We identified 354 474 individuals as incident new‐users of SGLT‐2 inhibitors and/or GLP‐1 RAs. After applying exclusion criteria, the base cohort comprised 220 043 individuals. Of these, 2660 individuals were incident new‐users of both SGLT‐2 inhibitors and GLP‐1 RAs. Among individuals who continued SGLT‐2 inhibitor therapy, we identified 1 379 193 dispensings from 217 383 individuals. Of these, 22 781 individuals initiated a GLP‐1 RA during continued SGLT‐2 inhibitor therapy (Figure 1).

After exclusions, the study cohort consisted of 24 765 combination therapy users (incident and prevalent new‐users) and 1 374 213 potential reference dispensings from SGLT‐2 inhibitor continuers. Before matching, combination therapy users were younger (mean age 62.8 vs. 68.2 years), were more frequently obese (64.5% vs. 43.7%), and had been continuously exposed to SGLT‐2 inhibitors for a mean of 1.6 years before initiating a GLP‐1 RA (see Table S4). Figure S2 shows the distribution of the time‐conditional propensity scores before and after matching for both treatment groups.

For 201 combination therapy users, no suitable reference dispensing could be identified for assignment to their corresponding hybrid exposure set. Additionally, 1644 hybrid exposure sets were excluded due to positivity violations, and no match could be found for 1256 combination therapy users. After matching, the final study cohort comprised 21 664 matched pairs with well‐balanced baseline covariates (see Table 1).

**TABLE 1:** Characteristics and corresponding absolute standardized differences (%) for combination therapy with SGLT‐2 inhibitors and GLP‐1 RAs compared with continued SGLT‐2 inhibitor therapy after matching on hybrid exposure sets and time‐conditional propensity scores.

Characteristic	Combination therapy (SGLT‐2 inhibitors and GLP‐1 RAs)	Continued SGLT‐2 inhibitor therapy	ASD (%)
People, *n*	21 664	21 664	
Age, in years, mean (SD)	63.3 (11.0)	63.3 (11.6)	0.81
Female, *n* (%)	9988 (46.1)	9952 (45.9)	0.33
Duration of diabetes, in years, mean (SD)	9.8 (5.3)	9.8 (5.3)	0.53
Diabetes‐related complications, *n* (%)			
Nephropathy	3183 (14.7)	3250 (15.0)	0.87
Retinopathy	2052 (9.5)	2100 (9.7)	0.75
Polyneuropathy	6129 (28.3)	6174 (28.5)	0.46
Foot syndrome	4362 (20.1)	4437 (20.5)	0.86
Comorbidities, *n* (%)			
Renal failure	5032 (23.2)	5093 (23.5)	0.67
Stroke	1342 (6.2)	1328 (6.1)	0.27
Angina pectoris	1111 (5.1)	1087 (5.0)	0.50
Myocardial infarction	1541 (7.1)	1475 (6.8)	1.20
Acute ischemic heart disease	179 (0.8)	177 (0.8)	0.10
Chronic ischemic heart disease	6237 (28.8)	6114 (28.2)	1.26
Heart failure	4177 (19.3)	4124 (19.0)	0.62
Atherosclerosis of extremities	1779 (8.2)	1783 (8.2)	0.07
Peripheral vascular disease	1047 (4.8)	1044 (4.8)	0.06
Hypertension	19 190 (88.6)	19 220 (88.7)	0.44
COPD	2775 (12.8)	2790 (12.9)	0.21
Osteoporosis	980 (4.5)	949 (4.4)	0.69
Dyslipidaemia	14 212 (65.6)	14 309 (66)	0.94
Cancer (excluding non‐melanoma skin cancer)	2558 (11.8)	2541 (11.7)	0.24
Nicotine dependency	3748 (17.3)	3738 (17.3)	0.12
Obesity	13 611 (62.8)	13 619 (62.9)	0.08
Depression and anxiety‐related disorders	9031 (41.7)	8924 (41.2)	1.00
Drug abuse	267 (1.2)	287 (1.3)	0.82
Sleep apnoe	500 (2.3)	470 (2.2)	0.94
Pancreatitis	345 (1.6)	353 (1.6)	0.29
Procedures, *n* (%)			
Revascularization	99 (0.5)	93 (0.4)	0.42
Coronary bypass/STENT	945 (4.4)	923 (4.3)	0.50
Bariatric surgery	58 (0.3)	51 (0.2)	0.65
Co‐medication, *n* (%)			
Benzodiazepines	541 (2.5)	492 (2.3)	1.48
Antidepressants	3801 (17.5)	3835 (17.7)	0.41
Opioids	2584 (11.9)	2522 (11.6)	0.89
Anticonvulsants	1580 (7.3)	1540 (7.1)	0.71
ACE inhibitors	8422 (38.9)	8445 (39.0)	0.22
Angiotensin II receptor blockers	9018 (41.6)	9036 (41.7)	0.17
Beta blockers	11 949 (55.2)	11 974 (55.3)	0.23
Loop diuretics	4820 (22.2)	4745 (21.9)	0.83
Other diuretics	4338 (20.0)	4341 (20.0)	0.03
Anti‐thrombotic agents	7127 (32.9)	7092 (32.7)	0.34
Calcium channel blockers	6598 (30.5)	6695 (30.9)	0.97
Corticosteroids	124 (0.6)	118 (0.5)	0.37
Bisphosphonates	164 (0.8)	142 (0.7)	1.21
Statins	11 928 (55.1)	12 057 (55.7)	1.20
Fibrates	345 (1.6)	347 (1.6)	0.07
Systemic corticosteroids	2029 (9.4)	1986 (9.2)	0.68
Glucose‐lowering drugs, *n* (%)			
Metformin	17 952 (82.9)	17 975 (83.0)	0.28
Alpha‐glucosidase inhibitors	61 (0.3)	50 (0.2)	1.00
Sulfonylureas	1987 (9.2)	2069 (9.6)	1.30
DPP‐4 inhibitors	10 533 (48.6)	10 508 (48.5)	0.23
Insulin and analogs	7172 (33.1)	7088 (32.7)	0.83
Total dispensations of DPP‐4 inhibitors and sulfonylureas, mean (SD)	1.9 (2.2)	1.9 (2.2)	0.04
Total dispensations of insulin and analogs, mean (SD)	2.0 (3.9)	1.9 (3.8)	1.21
Years of continued SGLT‐2 inhibitor therapy, mean (SD)	1.3 (1.5)	1.3 (1.5)	0.04
Health‐seeking behaviour, *n* (%)			
Influenca vaccination	8605 (39.7)	8730 (40.3)	1.18
Breast cancer screening	623 (2.9)	617 (2.8)	0.17
Neoplasm screening for men	2837 (13.1)	2863 (13.2)	0.36
Colonoscopy	124 (0.6)	117 (0.5)	0.43
Neoplasm screening for women	1339 (6.2)	1333 (6.2)	0.12
Skin cancer screening	1538 (7.1)	1494 (6.9)	0.80
Cohort entry year, *n* (%)			
2013	68 (0.3)	68 (0.3)	
2014	142 (0.7)	142 (0.7)	
2015	497 (2.3)	497 (2.3)	
2016	788 (3.6)	788 (3.6)	
2017	1093 (5.0)	1093 (5.0)	
2018	1419 (6.6)	1419 (6.6)	
2019	2123 (9.8)	2123 (9.8)	
2020	2742 (12.7)	2742 (12.7)	
2021	3812 (17.6)	3812 (17.6)	
2022	4448 (20.5)	4448 (20.5)	
2023	4532 (20.9)	4532 (20.9)	
Hospital admissions, *n* (%)			
0	14 973 (69.1)	15 043 (69.4)	0.70
1	4190 (19.3)	4136 (19.1)	0.63
2	1491 (6.9)	1491 (6.9)	0.00
≥3	1010 (4.7)	994 (4.6)	0.35
Outpatient admissions, *n* (%)			
≥1 and ≤10	4418 (20.4)	4324 (20.0)	1.08
≥11 and ≤20	11 716 (54.1)	11 829 (54.6)	1.05
≥21 and ≤30	4498 (20.8)	4492 (20.7)	0.07
>30	1032 (4.8)	1019 (4.7)	0.28
Federal state, *n* (%)			
Unknown	63 (0.3)	61 (0.3)	0.17
Schleswig‐Holstein	674 (3.1)	705 (3.3)	0.82
Hamburg (Hanseatic City)	233 (1.1)	223 (1.0)	0.45
Lower Saxony	1695 (7.8)	1658 (7.7)	0.64
Bremen (Hanseatic City)	27 (0.1)	26 (0.1)	0.13
North Rhine‐Westphalia	5263 (24.3)	5220 (24.1)	0.46
Hesse	1870 (8.6)	1848 (8.5)	0.36
Rhineland‐Palatinate	1157 (5.3)	1140 (5.3)	0.35
Bade‐Württemberg	1320 (6.1)	1365 (6.3)	0.86
Bavaria (Free State)	2559 (11.8)	2587 (11.9)	0.40
Saarland	280 (1.3)	283 (1.3)	0.12
Berlin	928 (4.3)	967 (4.5)	0.88
Brandenburg	1369 (6.3)	1315 (6.1)	1.03
Mecklenburg‐Western Pomerania	1010 (4.7)	1038 (4.8)	0.61
Saxony (Free State)	1161 (5.4)	1197 (5.5)	0.73
Saxony‐Anhalt	1116 (5.2)	1098 (5.1)	0.38
Thuringia (Free State)	939 (4.3)	933 (4.3)	0.14

Abbreviations: ACE, angiotensin‐converting enzyme; ASD, absolute standardized difference; COPD, chronic obstructive pulmonary disease; DPP‐4, dipeptidyl peptidase 4; GLP‐1 RA, glucagon‐like peptide‐1 receptor agonist; SD, standard deviation; SGLT‐2, sodium–glucose cotransporter 2.

Among combination therapy users, the most frequently prescribed combinations were dulaglutide with empagliflozin (25.0%), dulaglutide with dapagliflozin (24.1%), semaglutide with dapagliflozin (17.7%), and semaglutide with empagliflozin (14.4%). The median follow‐up time was 1.3 years (interquartile range 2.0 years).

### 3.1 Primary outcome

**FIGURE 2:**
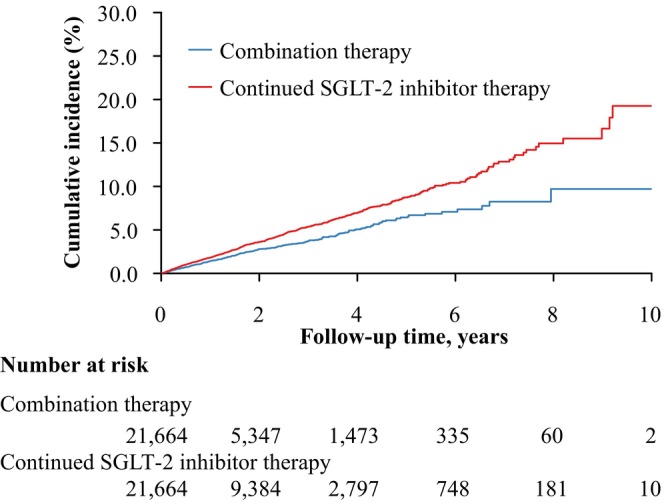
Cumulative incidence curves for all‐cause mortality comparing combination therapy with SGLT‐2 inhibitors and GLP‐1 RAs versus continued SGLT‐2 inhibitor therapy. Curves are based on the matched study cohort, with follow‐up starting from the study cohort index date. The red solid line represents continued SGLT‐2 inhibitor therapy, and the blue solid line represents combination therapy with SGLT‐2 inhibitors and GLP‐1 RAs. GLP‐1 RA, glucagon‐like peptide‐1 receptor agonists; SGLT‐2, sodium–glucose cotransporter 2.

Combination therapy users had a 29% lower hazard of all‐cause mortality compared with SGLT‐2 inhibitor continuers (410 vs. 868 events; 13.4 vs. 18.6 per 1000 person‐years; HR 0.71, 95% CI 0.63–0.80). The RD was −0.4% (95% CI −0.7% to −0.1%) at 1 year, increasing to −2.3% (95% CI −3.7% to −1.0%) at 5 years. Figure 2 shows the corresponding cumulative incidence curves for both treatment groups, and annual RDs up to 5 years are provided in Table S5.

### 3.2 Secondary outcomes

Combination therapy users had a 19% lower hazard for the modified cardiovascular composite outcome (740 vs. 1372 events; HR 0.81, 95% CI 0.74–0.88) and a 22% lower hazard for heart failure (304 vs. 562 events; HR 0.78, 95% CI 0.68–0.89) compared with SGLT‐2 inhibitor continuers. For myocardial infarction and stroke, combination therapy was associated with a 5% (HR 0.95, 95% CI 0.79–1.14) and 14% (HR 0.86, 95% CI 0.72–1.04) hazard reduction, respectively, though both confidence intervals included the null. Regarding renal outcomes, hazards were increased for nephropathy (HR 1.23, 95% CI 0.76–1.99) but 9% lower for renal failure (HR 0.91, 95% CI 0.70–1.17), again with wide confidence intervals. All outcomes are summarized in Table 2, with cumulative incidence curves provided in Figures S3–S8.

**TABLE 2:** Results for the primary and secondary outcomes for combination therapy with SGLT‐2 inhibitors and GLP‐1 RAs compared with continued SGLT‐2 inhibitor therapy after matching on hybrid exposure set and time‐conditional propensity scores.

Outcome	Individuals, *n*	Events	Person‐years	IR^a^ (95% CI)	HR^b^ (95% CI)
Primary outcome					
All‐cause mortality					
Combination therapy (SGLT‐2 inhibitors and GLP‐1 RAs)	21 664	410	30 660	13.4 (12.1–14.7)	0.71 (0.63–0.80)
Continued SGLT‐2 inhibitor therapy	21 664	868	46 626	18.6 (17.4–19.9)	Reference
Secondary outcomes					
Modified cardiovascular composite					
Combination therapy (SGLT‐2 inhibitors and GLP‐1 RAs)	21 664	740	30 279	24.4 (22.7–26.3)	0.81 (0.74–0.88)
Continued SGLT‐2 inhibitor therapy	21 664	1372	45 877	29.9 (28.3–31.5)	Reference
Myocardial infarction					
Combination therapy (SGLT‐2 inhibitors and GLP‐1 RAs)	21 664	190	30 447	6.2 (5.4–7.2)	0.95 (0.79–1.14)
Continued SGLT‐2 inhibitor therapy	21 664	302	46 264	6.5 (5.8–7.3)	Reference
Stroke					
Combination therapy (SGLT‐2 inhibitors and GLP‐1 RAs)	21 664	187	30 488	6.1 (5.3–7.1)	0.86 (0.72–1.04)
Continued SGLT‐2 inhibitor therapy	21 664	319	46 234	6.9 (6.2–7.7)	Reference
Heart failure					
Combination therapy (SGLT‐2 inhibitors and GLP‐1 RAs)	21 664	304	30 421	10.0 (8.9–11.2)	0.78 (0.68–0.89)
Continued SGLT‐2 inhibitor therapy	21 664	562	46 073	12.2 (11.2–13.2)	Reference
Nephropathy					
Combination therapy (SGLT‐2 inhibitors and GLP‐1 RAs)	21 664	32	30 629	1.0 (0.7–1.5)	1.23 (0.76–1.99)
Continued SGLT‐2 inhibitor therapy	21 664	36	46 600	0.8 (0.5–1.1)	Reference
Renal failure					
Combination therapy (SGLT‐2 inhibitors and GLP‐1 RAs)	21 664	99	30 607	3.2 (2.6–3.9)	0.91 (0.70–1.17)
Continued SGLT‐2 inhibitor therapy	21 664	157	46 538	3.4 (2.9–3.9)	Reference

Abbreviations: CI, confidence interval; GLP‐1 RA, glucagon‐like peptide‐1 receptor agonist; HR, hazard ratio; IR, incidence rate; SGLT‐2, sodium–glucose cotransporter 2.

^a^ Per 1000 person‐years.

^b^ Each model was 1:1 time‐conditional propensity score matched on hybrid exposure set and nearest neighbour.

### 3.3 Subgroup and sensitivity analyses

**FIGURE 3:**
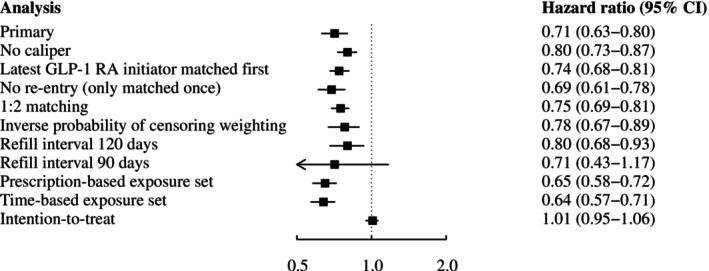
Forest plot showing hazard ratios for the primary outcome all‐cause mortality comparing combination therapy with SGLT‐2 inhibitors and GLP‐1 RAs versus continued SGLT‐2 inhibitor therapy, including the primary analysis and each sensitivity analysis. Error bars represent 95% confidence intervals. Figure adapted from Noh et al.^17^ GLP‐1 RA, glucagon‐like peptide‐1 receptor agonists; SGLT‐2, sodium–glucose cotransporter 2; CI, confidence interval.

No effect measure modification was observed across the examined subgroups (details provided in Tables S6‐S7). Stratified analyses showed comparable protective effects by sex (women: HR 0.64, 95% CI 0.52–0.78; men: HR 0.75, 95% CI 0.65–0.87) and baseline CVD status (history of CVD: HR 0.68, 95% CI 0.59–0.78; without history of CVD: HR 0.77, 95% CI 0.61–0.96). The hazard ratio remained robust across alternative matching procedures, including no caliper (HR 0.80, 95% CI 0.73–0.87), matching the latest GLP‐1 RA initiator first (HR 0.74, 95% CI 0.68–0.81), a one‐to‐two matching ratio (HR 0.75, 95% CI 0.69–0.81), and when excluding individuals who were initially matched as SGLT‐2 inhibitor continuers and later re‐entered the cohort as combination therapy users(HR 0.69, 95% CI 0.61–0.78). To address informative censoring, stabilized time‐varying IPCW revealed similar effects (HR 0.78, 95% CI 0.67–0.89). Altering the fixed refill interval to 120 days (HR 0.80, 95% CI 0.68–0.93) or 90 days (HR 0.71, 95% CI 0.43–1.17) yielded consistent findings, while an ITT exposure definition resulted in no differential effect (HR 1.01, 95% CI 0.95–1.06). Finally, prescription‐based (HR 0.65, 95% CI 0.58–0.72) and time‐based exposure sets (HR 0.64, 95% CI 0.57–0.71) were nearly identical to the original hybrid exposure set approach. A forest plot summarizing the hazard ratios for the primary outcome and all sensitivity analyses is presented in Figure 3.

## 4 DISCUSSION

In this large, real‐world cohort, combination therapy with SGLT‐2 inhibitors and GLP‐1 RAs was associated with a 29% lower hazard of all‐cause mortality compared with continuing SGLT‐2 inhibitors. This benefit was consistent across subgroups and supported by sensitivity analyses. Among secondary outcomes, combination therapy was associated with lower hazards for the modified cardiovascular composite (HR 0.81) and heart failure (HR 0.78). For myocardial infarction (HR 0.95), stroke (HR 0.86), and renal failure (HR 0.91), point estimates were directionally favourable, whereas diabetic nephropathy showed an increased hazard (HR 1.23). However, for these latter outcomes, confidence intervals were wide and included the null, indicating uncertainty and imprecision.

For the primary outcome of all‐cause mortality, the on‐treatment analysis showed a pronounced hazard reduction (HR 0.71). This analysis required concurrent exposure to both drug classes and estimates the effect of sustained, concurrent combination therapy compared with continued SGLT‐2 inhibitor therapy. In contrast, the ITT analysis followed individuals regardless of adherence and showed no mortality benefit (HR 1.01). Because the ITT analysis does not account for actual drug exposure, it likely diluted the effect by including individuals who may have switched to GLP‐1 RAs rather than maintaining combination therapy.

Our findings align with UK data showing a 27% lower risk of all‐cause mortality (HR 0.73, 95% CI 0.52–1.01) in individuals who, for the most part, had poor glycaemic control (HbA1c >8%).^9^ Importantly, we observed similar benefits in a German population with generally better glycaemic control,^18^ suggesting the advantages of combination therapy may extend beyond individuals with poorly controlled diabetes. Mortality benefits have also been reported using various designs and comparators—including SGLT‐2 inhibitors in post‐myocardial infarction,^19^ GLP‐1 RA plus sulfonylureas in US claims data,^20^ and metformin plus sulfonylureas in Danish registry data.^21^ Additionally, a study using US data reported consistent benefits across 3‐ and 5‐point major adverse cardiovascular events (HR 0.54 and 0.55, respectively).^22^ While that study matched randomly assigned index dates for the SGLT‐2 inhibitor comparator group to the distribution of time until GLP‐1 RA initiation, it could not account for time‐varying characteristics—a key advantage of the prevalent new‐user design.

Unlike the UK study's estimate for myocardial infarction (HR 0.73, 95% CI 0.48–1.12) and stroke (HR 0.86, 95% CI 0.46–1.59),^8^ our estimate for myocardial infarction was closer to the null (HR 0.95, 95% CI 0.79–1.14), whereas our estimate for stroke was almost identical (HR 0.86, 95% CI 0.72–1.04). For diabetic nephropathy, the increased hazard among combination therapy users (HR 1.23, 95% CI 0.76–1.99) may reflect prothopathic bias^23^ where GLP‐1 RA is initiated in response to persistent albuminuria.^7^ Interestingly, the more distal outcome, renal failure, showed a trend toward a lower hazard (HR 0.91, 95% CI 0.70–1.17), although both confidence intervals were wide and included the null.

### 4.1 Potential mechanisms

The observed benefits likely stem from the complementary mechanisms of both drug classes. SGLT‐2 inhibitors block glucose reabsorption in the proximal tubule, promoting urinary glucose excretion.^24^ Potential cardiorenal protection is driven by natriuresis and osmotic diuresis,^25^ reduced systolic blood pressure and cardiac pre‐ and afterload, improved cardiac metabolism,^26^ and modulation of the estimated glomerular filtration rate.^27^

In contrast, GLP‐1 RAs enhance glucose‐dependent insulin secretion and inhibit glucagon release. They reduce postprandial glucose and insulin concentrations by delaying gastric emptying.^28^ Furthermore, while SGLT‐2 inhibitors induce weight loss via caloric excretion and fluid reduction,^29^ GLP‐1 RAs promote weight loss through central nervous system pathways that increase satiety and reduce caloric intake.^5^ GLP‐1 RAs also improve LDL profiles^30^ and may inhibit atherosclerotic plaque progression.^31^ Targeting these distinct pathways of cardiorenal pathophysiology may explain the observed benefits.

While a meta‐analysis shows that SGLT‐2 inhibitors maintain cardiovascular benefits regardless of background GLP‐1 RA use,^27^ it was limited by a small sample size and a focus on baseline use of GLP‐1 RA, rather than sequential initiation.

### 4.2 Strengths and Limitations

Our study has several notable strengths. First, we selected continuation of SGLT‐2 inhibitor therapy as the comparator to reflect contemporary guideline‐supported treatment pathways, in which both SGLT‐2 inhibitors and GLP‐1 RAs are recommended and may be used in combination, whereas DPP‐4 inhibitors are not recommended for use alongside GLP‐1 RAs.^2^ Second, the prevalent new‐user design captured SGLT‐2 inhibitor users who later initiated GLP‐1 RA, reflecting real‐world prescribing patterns more comprehensively than traditional designs.^13^

Third, the use of hybrid exposure sets enabled precise matching on treatment history, reducing bias introduced by differences in prior treatment history. Fourth, the large, unselected real‐world population allowed us to examine effectiveness in a real‐world cohort, avoiding restrictive eligibility criteria common in clinical trials.^32^

Nevertheless, some limitations must be acknowledged. Outcome definitions based on health claims data used in this study may be subject to bias, as no external validation studies exist. However, we restricted outcomes to hospital diagnoses in primary position to enhance validity. Residual confounding remains a concern, as individuals initiating combination therapy may systematically differ from those continuing SGLT‐2 inhibitors. We lacked factors such as HbA1c, BMI, albuminuria, lifestyle, and socioeconomic status. While we used markers of health‐seeking behaviour as proxies for socioeconomic status, these differences may still contribute to the observed associations, despite the prevalent new‐user design and extensive covariate adjustment.

However, the consistent protective effect across subgroups and sensitivity analyses alongside comparable results from the UK study^9^ that accounted for HbA1c and BMI supports the robustness of our findings, although caution is still warranted.

Further, on‐treatment estimates are susceptible to informative censoring, as individuals who discontinue therapy may differ in their risk of the outcome compared with those who continue therapy.^23^ Since combination therapy required two refills versus one for SGLT‐2 inhibitor continuers, the resulting differential follow‐up times, as well as differences in treatment persistence and healthy adherer behaviour, could have influenced our results. To address informative censoring, we applied stabilized time‐varying IPCW, which yielded nearly identical results (HR 0.78, 95% CI 0.67–0.89).

Moreover, the impact of therapy sequence is unclear. In future, assessment of the reversed sequence—compared with continued GLP‐1 RA therapy—may clarify whether benefits depend on sequence or on combination therapy per se.

## 5 CONCLUSION

In conclusion, our findings provide evidence that combination therapy is associated with a reduced hazard of all‐cause mortality, the modified cardiovascular composite outcome, and heart failure in a broad population of individuals with type 2 diabetes. While point estimates for myocardial infarction, stroke, and renal failure were suggestive of a protective effect, these results and the observed increased hazard for diabetic nephropathy lacked statistical precision. Overall, these results suggest that sustained concurrent use of both drug classes may offer meaningful clinical advantages in routine practice, though the findings for specific secondary outcomes warrant further investigation.

## AUTHOR CONTRIBUTIONS

G.M., O.K., and W.R. were involved in the conception, design, and conduct of the study, as well as in the analysis and interpretation of the results. G.M. prepared the data and conducted the statistical analyses together with O.K. B.H. was responsible for data provision and curation. G.M. wrote the first draft of the manuscript, and all authors edited, reviewed, and approved the final version of the manuscript. G.M. and O.K. are the guarantors of this work and, as such, had full access to all the data in the study and take responsibility for the integrity of the data and the accuracy of the data.

## FUNDING INFORMATION

The German Diabetes Center is funded by the German Federal Ministry of Health, the Ministry of Culture and Science of the State of North Rhine‐Westphalia, and grants from the German Federal Ministry of Education and Research to the German Center for Diabetes Research (DZD). The funding source had no role in the design of the study, analysis of the results, writing of the manuscript, or decision to submit for publication.

## CONFLICT OF INTEREST STATEMENT

W.R. reports the receipt of consulting fees for attending educational sessions by Novo Nordisk outside of the topic of the current work. O.K. received honoraria for biostatistical education from Berlin‐Chemie. G.M. and B.H. have nothing to disclose.

## ETHICS STATEMENT

The study was approved by the Ethics Committee of the Medical Faculty of Heinrich Heine University Düsseldorf, Germany (approval number [2025‐3289, 2025‐3289_1]).

## Supporting information


**Figure S1.** Graphical depiction of the hybrid exposure sets and possible matching timepoints.
**Figure S2.** Distribution of the time‐conditional propensity scores before matching (A) and after matching (B) for combination therapy users of SGLT‐2 inhibitors and GLP‐1 RAs and continuers of SGLT‐2 inhibitor therapy.
**Figure S3.** Cumulative incidence curves for the modified cardiovascular composite comparing combination therapy with SGLT‐2 inhibitors and GLP‐1 RAs versus continued SGLT‐2 inhibitor therapy.
**Figure S4.** Cumulative incidence curves for heart failure comparing combination therapy with SGLT‐2 inhibitors and GLP‐1 RAs versus continued SGLT‐2 inhibitor therapy.
**Figure S5.** Cumulative incidence curves for myocardial infarction comparing combination therapy with SGLT‐2 inhibitors and GLP‐1 RAs versus continued SGLT‐2 inhibitor therapy.
**Figure S6.** Cumulative incidence curves for stroke comparing combination therapy with SGLT‐2 inhibitors and GLP‐1 RAs versus continued SGLT‐2 inhibitor therapy.
**Figure S7.** Cumulative incidence curves for diabetic nephropathy comparing combination therapy with SGLT‐2 inhibitors and GLP‐1 RAs versus continued SGLT‐2 inhibitor therapy.
**Figure S8.** Cumulative incidence curves for renal failure comparing combination therapy with SGLT‐2 inhibitors and GLP‐1 RAs versus continued SGLT‐2 inhibitor therapy.
**Table S1.** Definition of type 2 diabetes.
**Table S2.** Definition of covariates.
**Table S3.** Definition of study outcomes.
**Table S4.** Characteristics and corresponding absolute standardized differences (%) for combination therapy with SGLT‐2 inhibitors and GLP‐1 RAs compared with continued SGLT‐2 inhibitor therapy before matching on hybrid exposure sets and time‐conditional propensity scores.
**Table S5.** Risk differences for the primary outcome all‐cause mortality comparing combination therapy with SGLT‐2 inhibitors and GLP‐1 RAs versus continued SGLT‐2 inhibitor therapy.
**Table S6.** Hazard ratios by sex for the primary outcome all‐cause mortality comparing combination therapy with SGLT‐2 inhibitors and GLP‐1 RAs versus continued SGLT‐2 inhibitor therapy.
**Table S7.** Hazard ratios by cardiovascular disease status for the primary outcome all‐cause mortality comparing combination therapy with SGLT‐2 inhibitors and GLP‐1 RAs versus continued SGLT‐2 inhibitor therapy.

## Data Availability

The datasets analysed in this study are not publicly available due to data protection regulations and contractual agreements with the data provider, BARMER (Wuppertal, Germany).
