# Supplementary material for: Comparative effectiveness of combination therapy with SGLT‐2 inhibitors and GLP‐1 RAs compared with SGLT‐2 inhibitors in individuals with type 2 diabetes: A prevalent new‐user cohort study
Source: Diabetes Obes Metab. 2026 Feb 22;28(4):3273–84. doi: 10.1111/dom.70523 (PMC12992157; doi:10.1111/dom.70523)
Supplement: Supplementary file 1 — Figure S1. Graphical depiction of the hybrid exposure sets and possible matching timepoints. Figure S2. Distribution of the time‐conditional propensity scores before matching (A) and after matching (B) for combination therapy users of SGLT‐2 inhibitors and GLP‐1 RAs and continuers of SGLT‐2 inhibitor therapy. Figure S3. Cumulative incidence curves for the modified cardiovascular composite comparing combination therapy with SGLT‐2 inhibitors and GLP‐1 RAs versus continued SGLT‐2 inhibitor therapy. Figure S4. Cumulative incidence curves for heart failure comparing combination therapy with SGLT‐2 inhibitors and GLP‐1 RAs versus continued SGLT‐2 inhibitor therapy. Figure S5. Cumulative incidence curves for myocardial infarction comparing combination therapy with SGLT‐2 inhibitors and GLP‐1 RAs versus continued SGLT‐2 inhibitor therapy. Figure S6. Cumulative incidence curves for stroke comparing combination therapy with SGLT‐2 inhibitors and GLP‐1 RAs versus continued SGLT‐2 inhibitor therapy. Figure S7. Cumulative incidence curves for diabetic nephropathy comparing combination therapy with SGLT‐2 inhibitors and GLP‐1 RAs versus continued SGLT‐2 inhibitor therapy. Figure S8. Cumulative incidence curves for renal failure comparing combination therapy with SGLT‐2 inhibitors and GLP‐1 RAs versus continued SGLT‐2 inhibitor therapy. Table S1. Definition of type 2 diabetes. Table S2. Definition of covariates. Table S3. Definition of study outcomes. Table S4. Characteristics and corresponding absolute standardized differences (%) for combination therapy with SGLT‐2 inhibitors and GLP‐1 RAs compared with continued SGLT‐2 inhibitor therapy before matching on hybrid exposure sets and time‐conditional propensity scores. Table S5. Risk differences for the primary outcome all‐cause mortality comparing combination therapy with SGLT‐2 inhibitors and GLP‐1 RAs versus continued SGLT‐2 inhibitor therapy. Table S6. Hazard ratios by sex for the primary outcome all‐cause mortality comparing [file DOM-28-3273-s001.docx]

Comparative effectiveness of combination therapy with SGLT-2 inhibitors and GLP-1 RAs compared with SGLT-2 inhibitors in individuals with type 2 diabetes: a prevalent new-user cohort study

Gregor A. Maier MSc^1^, Beata Hennig Diplom (FH)^2^, Wolfgang Rathmann MD^1^, Oliver Kuss PhD^1,3,4^

Affiliations

^1^ Institute for Biometrics and Epidemiology, German Diabetes Center, Leibniz Center for Diabetes Research at Heinrich Heine University, Düsseldorf, Germany

^2^ Department of Medicine and Health Services Research, BARMER Health Insurance, Wuppertal, Germany

^3^ German Center for Diabetes Research, München-Neuherberg, Germany

^4^ Centre for Health and Society, Medical Faculty and University Hospital Düsseldorf, Heinrich Heine University Düsseldorf, Germany

Corresponding author:

Gregor A. Maier, MSc

German Diabetes Center,

Institute for Biometrics and Epidemiology,

Auf’m Hennekamp 65,

40225 Düsseldorf, Germany

E-Mail: [gregor.maier@ddz.de](mailto:gregor.maier@ddz.de),

Phone: +49-(0)-211-33-82-338

**CONTENT**

[Supplementary Figures 4](#_Toc221262383)

[Figure S1. Graphical representation of the hybrid exposure sets and possible matching timepoints 4](#_Toc221262384)

[Figure S2. Distribution of the time-conditional propensity scores before matching (A) and after matching (B) for combination therapy users of SGLT-2 inhibitors and GLP-1 RAs and continuers of SGLT-2 inhibitor therapy 6](#_Toc221262385)

[Figure S3. Cumulative incidence curves for the modified cardiovascular composite comparing combination therapy with SGLT-2 inhibitors and GLP-1 RAs versus continued SGLT-2 inhibitor therapy. 7](#_Toc221262386)

[Figure S4. Cumulative incidence curves for heart failure comparing combination therapy with SGLT-2 inhibitors and GLP-1 RAs versus continued SGLT-2 inhibitor therapy. 7](#_Toc221262387)

[Figure S5. Cumulative incidence curves for myocardial infarction comparing combination therapy with SGLT-2 inhibitors and GLP-1 RAs versus continued SGLT-2 inhibitor therapy. 8](#_Toc221262388)

[Figure S6. Cumulative incidence curves for stroke comparing combination therapy with SGLT-2 inhibitors and GLP-1 RAs versus continued SGLT-2 inhibitor therapy. 8](#_Toc221262389)

[Figure S7. Cumulative incidence curves for diabetic nephropathy comparing combination therapy with SGLT-2 inhibitors and GLP-1 RAs versus continued SGLT-2 inhibitor therapy. 9](#_Toc221262390)

[Figure S8. Cumulative incidence curves for renal failure comparing combination therapy with SGLT-2 inhibitors and GLP-1 RAs versus continued SGLT-2 inhibitor therapy. 9](#_Toc221262391)

[Supplementary Tables 10](#_Toc221262392)

[Table S1. Definition of type 2 diabetes 10](#_Toc221262393)

[Table S2. Definition of covariates 10](#_Toc221262394)

[Table S3. Definition of study outcomes 14](#_Toc221262395)

[Table S4. Characteristics and corresponding absolute standardized differences (%) for combination therapy with SGLT-2 inhibitors and GLP-1 RAs compared with continued SGLT-2 inhibitor therapy before matching on hybrid exposure sets and time-conditional propensity scores 14](#_Toc221262396)

[Table S5. Risk differences for the primary outcome all-cause mortality comparing combination therapy with SGLT-2 inhibitors and GLP-1 RAs versus continued SGLT-2 inhibitor therapy 17](#_Toc221262397)

[Table S6. Hazard ratios by sex for the primary outcome all-cause mortality comparing combination therapy with SGLT-2 inhibitors and GLP-1 RAs versus continued SGLT-2 inhibitor therapy 17](#_Toc221262398)

[Table S7. Hazard ratios by cardiovascular disease status for the primary outcome all-cause mortality comparing combination therapy with SGLT-2 inhibitors and GLP-1 RAs versus continued SGLT-2 inhibitor therapy 18](#_Toc221262399)

# **Supplementary Figures**

## **Figure S1.** Graphical representation of the hybrid exposure sets and possible matching timepoints


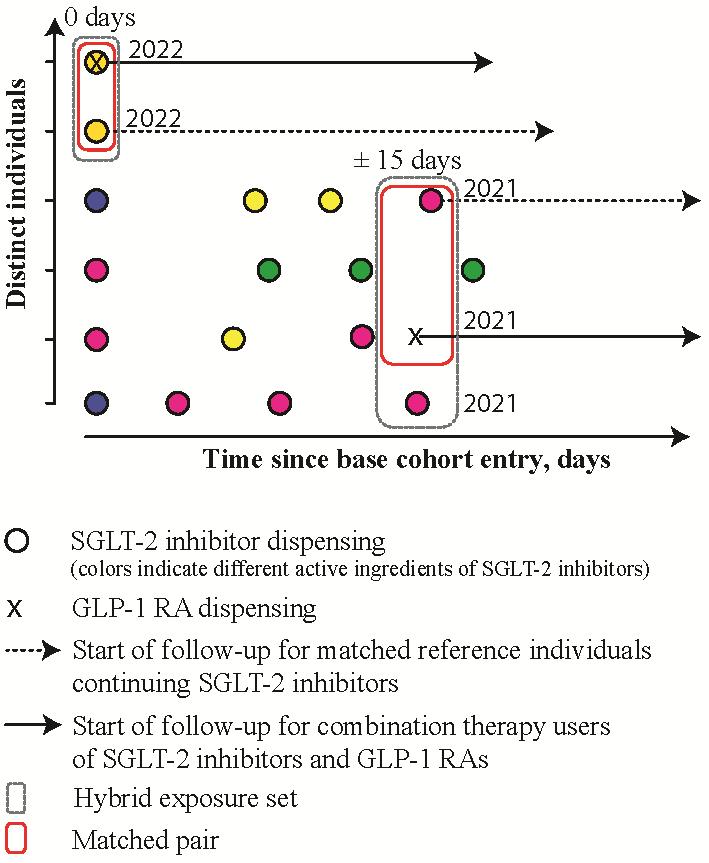


GLP-1 RA: Glucagon-like peptide-1 receptor agonists; SGLT-2: Sodium–glucose cotransporter 2

Figure adapted from Suissa et al. ^1^ and Simms-Williams et al. ^2^

For each combination therapy user (indicated by 'X' in Figure 1), a hybrid exposure set ^4^ (indicated by a dashed grey box in Figure 1) was defined to summarize the treatment history up to the timepoint of GLP-1 RA initiation. Each hybrid exposure set incorporated four key aspects: First, the cumulative number of SGLT-2 inhibitor drug dispensings received including the first GLP-1 RA dispensing (SGLT-2 inhibitor dispensings are represented as colored circles in Figure 1); for incident new-users of the combination therapy, this cumulative number was set to 1. Second, time in days since base cohort entry (incident new-use of an SGLT-2 inhibitor) until starting combination therapy (corresponding to 0 for incident new-users of combination therapy). Third, the specific active SGLT-2 inhibitor ingredient used most recently (indicated by the different colors in Figure 1). Fourth, the calendar year of the GLP-1 RA dispensing.
Each hybrid exposure set included all eligible reference dispensings of SGLT-2 inhibitors contributed by individuals who had not initiated GLP-1 RA up to that timepoint. To be eligible, a reference dispensing had to correspond **exactly to: the same sequential SGLT-2 inhibitor dispensing number** as the GLP-1 RA initiation of the combination therapy user, the duration of continuous SGLT-2 inhibitor therapy since base cohort entry (±15 days for prevalent new-users; 0 for incident new-users), the most recently dispensed SGLT-2 inhibitor active ingredient, and the calendar year of the respective dispensing.
The process of time-conditional propensity score (TCPS) calculation and subsequent TCPS matching is described in the main text. In Figure 1, a TCPS matched pair is indicated by the solid red box.
Consequently, in the matched dataset, the unit of interest shifts to the individual itself rather than their dispensings, as the dispensings were used solely to establish a comparable "starting point" that aligns with the timepoint of GLP-1 RA initiation of the respective combination therapy user. ^3^
Within each matched pair, the solid arrow illustrates the start of follow-up time for the combination therapy user, while the dotted arrow illustrates the start of follow-up time for the matched reference individual.

## **Figure S2.** Distribution of the time-conditional propensity scores before matching (A) and after matching (B) for combination therapy users of SGLT-2 inhibitors and GLP-1 RAs and continuers of SGLT-2 inhibitor therapy


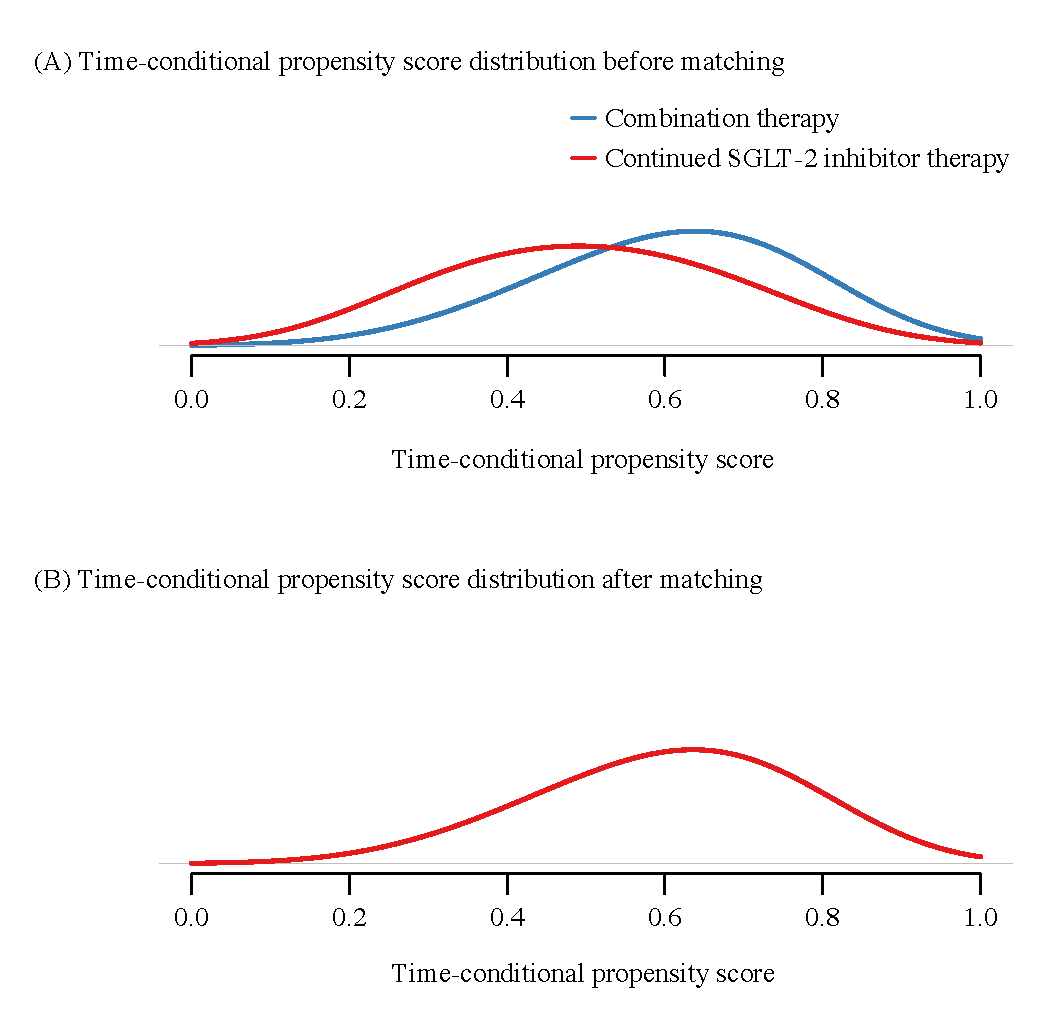


GLP-1 RA: Glucagon-like peptide-1 receptor agonists; SGLT-2: Sodium–glucose cotransporter 2

## **Figure S3.** Cumulative incidence curves for the modified cardiovascular composite comparing combination therapy with SGLT-2 inhibitors and GLP-1 RAs versus continued SGLT-2 inhibitor therapy.


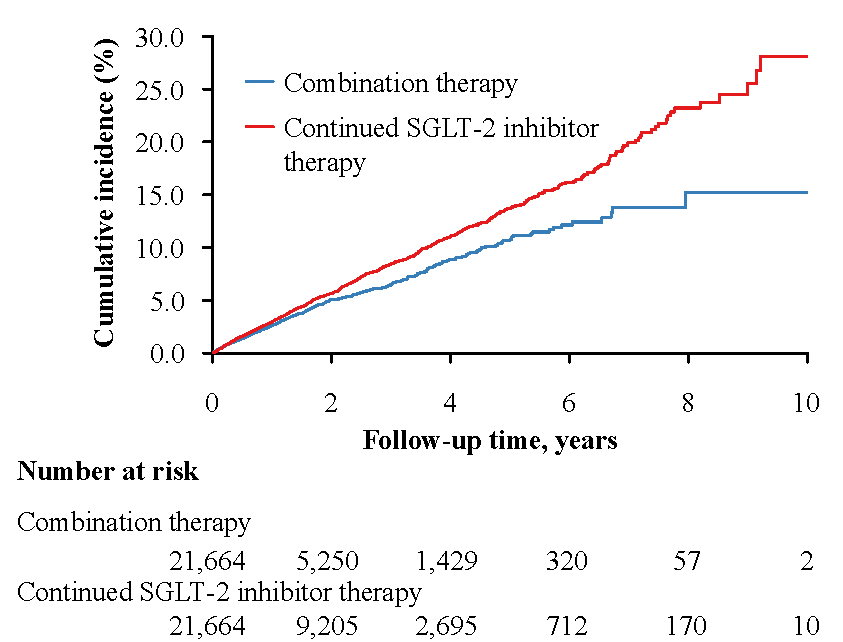


GLP-1 RA: Glucagon-like peptide-1 receptor agonists; SGLT-2: Sodium–glucose cotransporter 2

## **Figure S4.** Cumulative incidence curves for heart failure comparing combination therapy with SGLT-2 inhibitors and GLP-1 RAs versus continued SGLT-2 inhibitor therapy.


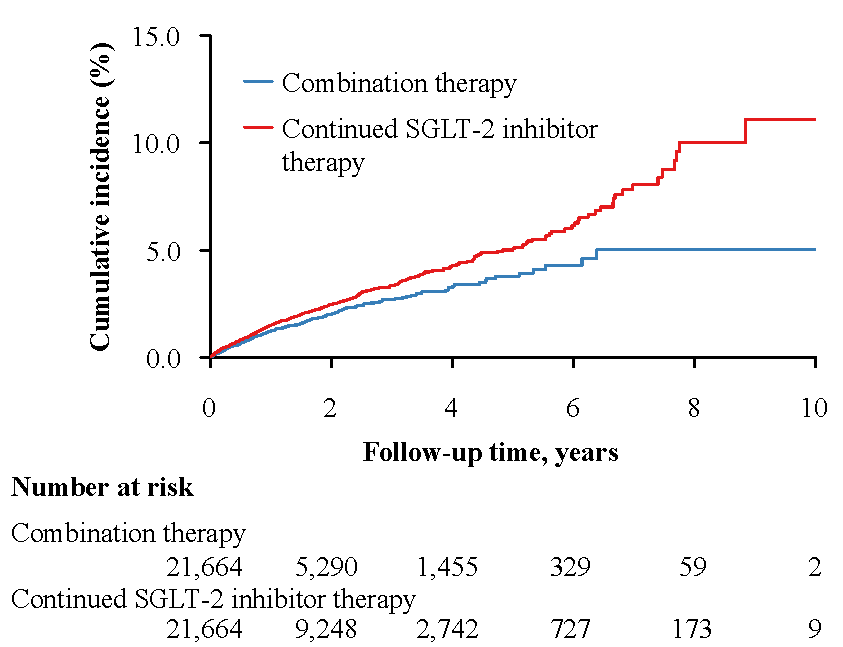


GLP-1 RA: Glucagon-like peptide-1 receptor agonists; SGLT-2: Sodium–glucose cotransporter 2

## **Figure S5.** Cumulative incidence curves for myocardial infarction comparing combination therapy with SGLT-2 inhibitors and GLP-1 RAs versus continued SGLT-2 inhibitor therapy.


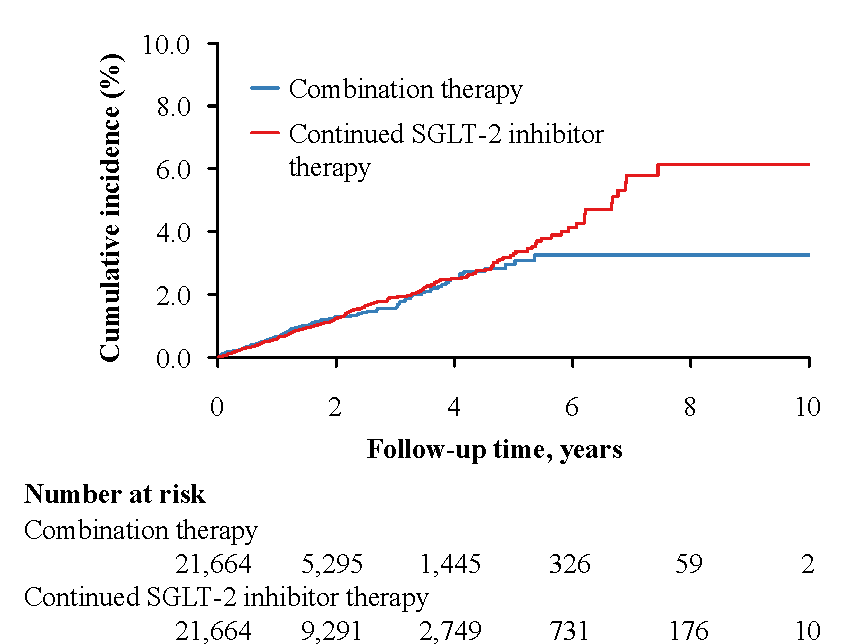


GLP-1 RA: Glucagon-like peptide-1 receptor agonists; SGLT-2: Sodium–glucose cotransporter 2

## **Figure S6.** Cumulative incidence curves for stroke comparing combination therapy with SGLT-2 inhibitors and GLP-1 RAs versus continued SGLT-2 inhibitor therapy.


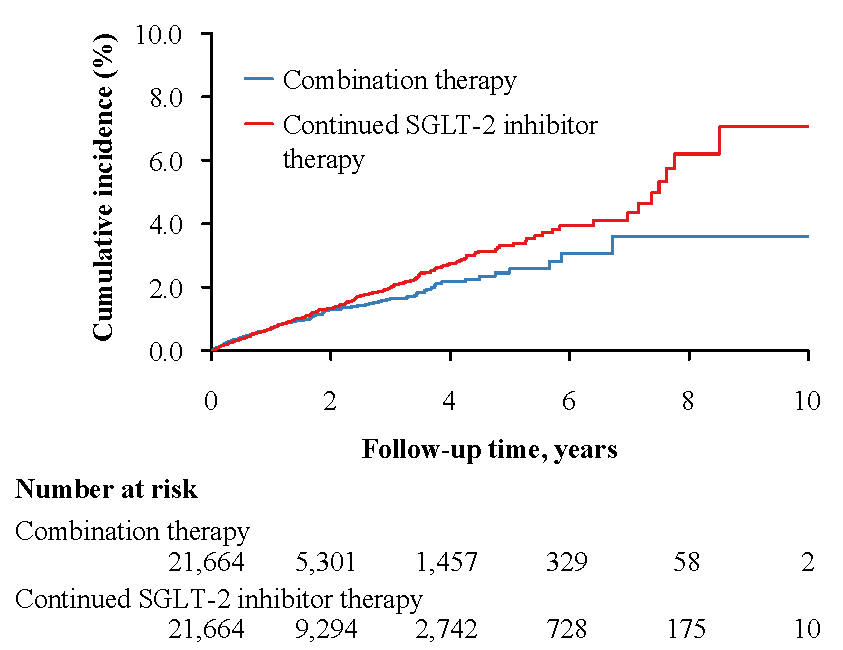


GLP-1 RA: Glucagon-like peptide-1 receptor agonists; SGLT-2: Sodium–glucose cotransporter 2

## **Figure S7.** Cumulative incidence curves for diabetic nephropathy comparing combination therapy with SGLT-2 inhibitors and GLP-1 RAs versus continued SGLT-2 inhibitor therapy.


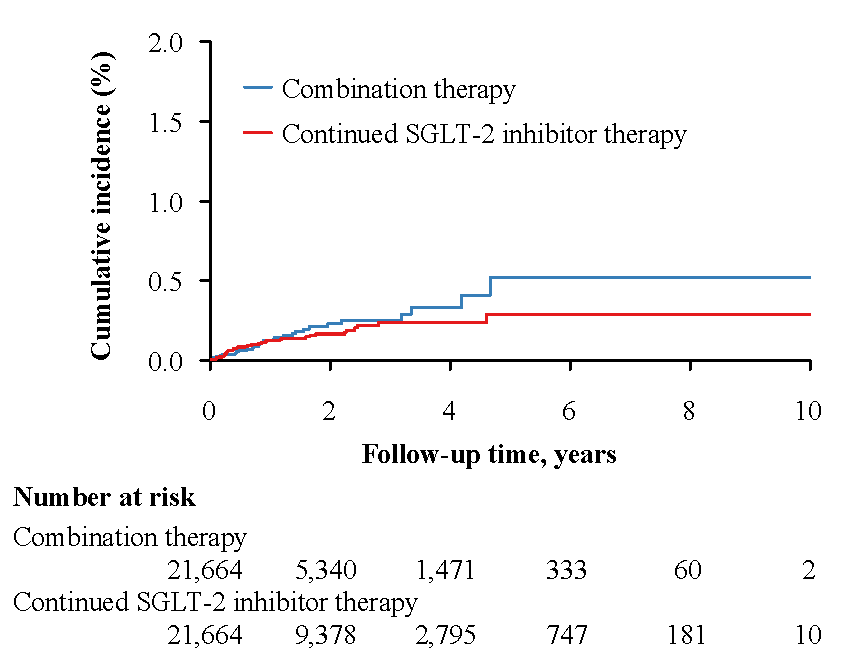


GLP-1 RA: Glucagon-like peptide-1 receptor agonists; SGLT-2: Sodium–glucose cotransporter 2

## **Figure S8.** Cumulative incidence curves for renal failure comparing combination therapy with SGLT-2 inhibitors and GLP-1 RAs versus continued SGLT-2 inhibitor therapy.


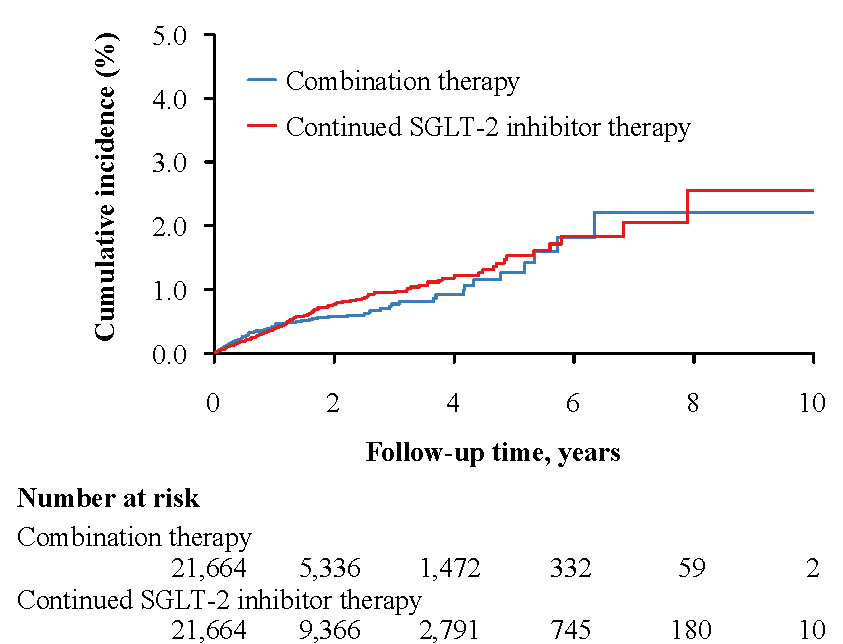


GLP-1 RA: Glucagon-like peptide-1 receptor agonists; SGLT-2: Sodium–glucose cotransporter 2

# **Supplementary Tables**

## **Table S1.** Definition of type 2 diabetes

| … | | | **code(s)** | | | |
| --- | --- | --- | --- | --- | --- | --- |
| **Type 2 diabetes** | **INSULIN** | **GLA** | **E10.x** | **E11.x/E12.x** | **E13.x** | **E14.x** |
| … | - | - | 0 | 1 | - | - |
| **OR** | - | 1 | 1 | 1 | - | - |
| **OR** | 0 | - | 1 | 1 | - | - |
| **OR** | - | 1 | 0 | 0 | 0 | 1 |

GLA: Glucose-lowering agent

A value of '1' indicates a diagnosis or dispensing exists, '0' indicates it does not exist, and '-' indicates it can either exist or not exist. Outpatient diagnoses must be coded as 'G' or 'Z' according to German coding guidelines. Inpatient diagnoses were considered as main diagnoses.

Individuals were excluded from the study if any of these defined combinations of diagnoses and/ or prescriptions did not exist before the base cohort index date. Table adapted from Reitzle et al. ^4^

## **Table S2.** Definition of covariates

|  | Code(s) | Assessment | Setting | Suffix | Reference |
| --- | --- | --- | --- | --- | --- |
| Age | Age at the time of study cohort entry | [0,0] | … | … | … |
| Sex | Sex at the time of study cohort entry | [0,0] | … | … | … |
| Duration of diabetes | In years, since first diagnosis | [-∞,0] | … | … | … |
| Diabetes-related complications | … | … | … | … | … |
| Nephropathy | E10-14x + .20, .21, .72, .73, .74, .75, N083x | [-365,0] | Outpatient, Inpatient | G, Z, main- and secondary diagnoses | ^5^ |
| Retinopathy | H360 OR (E1x3 AND (H350 OR H352 OR H358)) | [-365,0] | Outpatient, Inpatient | G, Z, main- and secondary diagnoses | ^6^ |
| Polyneuropathy | G632 OR ((E1x4 OR E1x74 OR E1x75) AND (G629 OR G633)) | [-365,0] | Outpatient, Inpatient | G, Z, main- and secondary diagnoses | ^6^ |
| Foot syndrome | E1x74 OR E1x75 OR M1427 OR M1467 | [-365,0] | Outpatient, Inpatient | G, Z, main- and secondary diagnoses | ^5^ |
| Comorbidities | … | … | … | … | … |
| Renal failure | N17x-N19x | [-365,0] | Outpatient, Inpatient | G, Z, main- and secondary diagnoses | … |
| Stroke | I60x-I64x | [-365,0] | Outpatient, Inpatient | G, Z, main- and secondary diagnoses | … |
| Angina pectoris | I20x | [-365,0] | Outpatient, Inpatient | G, Z, main- and secondary diagnoses | … |
| Myocardial infarction | I21x,I22x | [-365,0] | Outpatient, Inpatient | G, Z, main- and secondary diagnoses | … |
| Acute ischemic heart diseases | I24x | [-365,0] | Outpatient, Inpatient | G, Z, main- and secondary diagnoses | … |
| Chronic ischemic heart disease | I25x | [-365,0] | Outpatient, Inpatient | G, Z, main- and secondary diagnoses | … |
| Heart failure | I50x, I130, I110, I132 | [-365,0] | Outpatient, Inpatient | G, Z, main- and secondary diagnoses | … |
| Atherosclerosis of extremities | I702 | [-365,0] | Outpatient, Inpatient | G, Z, main- and secondary diagnoses | … |
| Peripheral vascular disease | I739 | [-365,0] | Outpatient, Inpatient | G, Z, main- and secondary diagnoses | … |
| Hypertension | I10x- I13x, I15x | [-365,0] | Outpatient, Inpatient | G, Z, main- and secondary diagnoses | … |
| COPD | J44x | [-365,0] | Outpatient, Inpatient | G, Z, main- and secondary diagnoses | … |
| Osteoporosis | M80x, M81x | [-365,0] | Outpatient, Inpatient | G, Z, main- and secondary diagnoses | … |
| Dyslipidemia | E78x | [-365,0] | Outpatient, Inpatient | G, Z, main- and secondary diagnoses | … |
| Cancer (excluding non-melanoma skin cancer) | C00x-C97x, except C44x | [-365,0] | Outpatient, Inpatient | G, Z, main- and secondary diagnoses | … |
| Nicotine dependency | F17x | [-365,0] | Outpatient, Inpatient | G, Z, main- and secondary diagnoses | … |
| Obesity | E66x | [-365,0] | Outpatient, Inpatient | G, Z, main- and secondary diagnoses | … |
| Depression and/ or anxiety-related disorders | F32x, F33x, F34x, F38x-F45x, F48x | [-365,0] | Outpatient, Inpatient | G, Z, main- and secondary diagnoses | … |
| Drug abuse | F11x-F16x, F18x, F19x, R78x, T40x | [-365,0] | Outpatient, Inpatient | G, Z, main- and secondary diagnoses | … |
| Sleep apnoe | G473x | [-365,0] | Outpatient, Inpatient | G, Z, main- and secondary diagnoses | … |
| Pancreatitis | K860, K861 | [-365,0] | Outpatient, Inpatient | G, Z, main- and secondary diagnoses | … |
| Procedures | … | … | … | … | … |
| Revascularization | 5-361x, 5-362x, 5-363x | [-365,0] | Outpatient, Inpatient | OPS | … |
| Coronary bypass / STENT | 8-836x, 8-837x, 8-84x | [-365,0] | Outpatient, Inpatient | OPS | … |
| Bariatric surgery | 5-434.3, 5-434.4, 5-434.5, 5-445.0, 5-445.1, 5-445.2, 5-445.4, 5-445.5, 5-448.a, 5-448.b, 5-448.c | [-∞,0] | Outpatient, Inpatient | OPS | … |
| Co-medications | … | … | … | … | … |
| Benzodiazepine | N05BAx | [-365,0] | Drug dispensation dates | … | … |
| Antidepressants | N06Ax | [-365,0] | Drug dispensation dates | … | … |
| Opioids | N02Ax | [-365,0] | Drug dispensation dates | … | … |
| Anticonvulsants | N03x | [-365,0] | Drug dispensation dates | … | … |
| ACE inhibitors | C09Ax, C09Bx | [-365,0] | Drug dispensation dates | … | … |
| Angiotensin II receptor blockers | C09Cx, C09Dx | [-365,0] | Drug dispensation dates | … | … |
| Beta blockers | C07x | [-365,0] | Drug dispensation dates | … | … |
| Loop diuretics | C03Cx | [-365,0] | Drug dispensation dates | … | … |
| Other diuretics | C03Ax, C03Bx, C03Dx | [-365,0] | Drug dispensation dates | … | … |
| Anti thrombotic agents | B01x | [-365,0] | Drug dispensation dates | … | … |
| Calcium channel blockers | C08x | [-365,0] | Drug dispensation dates | … | … |
| Corticosteroids | A01ACx | [-365,0] | Drug dispensation dates | … | … |
| Bisphosphonate | M05BAx, M05BBx | [-365,0] | Drug dispensation dates | … | … |
| Statins | C10AAx | [-365,0] | Drug dispensation dates | … | … |
| Fibrates | C10ABx | [-365,0] | Drug dispensation dates | … | … |
| Systemic corticosteroids | H02x | [-365,0] | Drug dispensation dates | … | … |
| Glucose-lowering drugs |  |  |  |  |  |
| Metformin | Dispensation dates | [-365,0] | Drug dispensation dates | … | … |
| Alpha glucosidase inhibitors | Dispensation dates | [-365,0] | Drug dispensation dates | … | … |
| Sulfonylureas | Dispensation dates | [-365,0] | Drug dispensation dates | … | … |
| DPP-4 inhibitors | Dispensation dates | [-365,0] | Drug dispensation dates | … | … |
| Insulin and analogs | Dispensation dates | [-365,0] | Drug dispensation dates | … | … |
| Total dispensations of DPP-4 inhibitors and sulfonylureas | Total dispensation dates | [-365,0] | Drug dispensation dates | … | … |
| Total dispensations of insulin and analogs | Total dispensation dates | [-365,0] | Drug dispensation dates | … | … |
| Total years of SGLT-2 inhibitor use | Total use in years since base cohort entry | [-∞,0] | Drug dispensation dates | … | … |
| Health-seeking behaviour | … | … | … | … | … |
| Influenza vaccination | 89111, 89112, 89112Y | [-365,0] | GOP | … | … |
| Breast cancer screening | 01750 | [-365,0] | GOP | … | … |
| Neoplasm screening for men | 01731 | [-365,0] | GOP | … | … |
| Colonoscopy | 01741 | [-365,0] | GOP | … | … |
| Neoplasm screening for women | 01760 | [-365,0] | GOP | … | … |
| Skin cancer screening | 01745, 01746 | [-365,0] | GOP | … | … |
| Cohort entry year | Year of cohort entry |  |  |  |  |
| Hospital admissions | Categorized in:  Zero,  One,  Two,  more than three hospital admissions one year prior study cohort index | [-365,0] | Inpatient | … | … |
| Outpatient admissions | Categorized in:  One to ten,  11–20,  21–30,  more than 30 | [-365,0] | Outpatient | … | … |
| Federal state | Residency at study cohort entry | [0,0] | … | … | … |

ACE: angiotensin-converting enzyme; COPD: chronic obstructive pulmonary disease; DPP-4: dipeptidyl peptidase 4; GOP: Gebührenordnungsposition; OPS: Operationen- und Prozedurenschlüssel

## **Table S3.** Definition of study outcomes

| … | **ICD-10-GM code(s)** | **Care setting** | **Suffix** |
| --- | --- | --- | --- |
| All-cause mortality | Date of death | … | … |
| Modified cardiovascular composite | Date of death, I60.x–I64.x, I21.x, I22.x | Inpatient | Primary |
| Hospitalization for myocardial infarction | I21.x, I22.x | Inpatient | Primary |
| Hospitalization for stroke | I60.x–I64.x | Inpatient | Primary |
| Hospitalization for heart failure | I50.x, I130, I110, I132 | Inpatient | Primary |
| Hospitalization for diabetic nephropathy | E10-14.x + .20, .21, .72, .73, .74, .75 primary, N083 | Inpatient | Primary |
| Hospitalization for renal failure | N17.x, N18.x, N19.x | Inpatient | Primary |

ICD-10-GM: International Classification of Diseases, tenth revision, German modification

## **Table S4.** Characteristics and corresponding absolute standardized differences (%) for combination therapy with SGLT-2 inhibitors and GLP-1 RAs compared with continued SGLT-2 inhibitor therapy before matching on hybrid exposure sets and time-conditional propensity scores

| **Characteristic** | **Combination therapy (SGLT-2 inhibitors and GLP-1 RAs)** | **Continued SGLT-2 inhibitor therapy** | **ASD (%)** |
| --- | --- | --- | --- |
| People, n | 24,765 | 1,374,213 |  |
|  |  |  |  |
| Age, in years, mean (SD) | 62.8 (11.2) | 68.2 (11.4) | 48.22 |
| Female, n (%) | 11,556 (46.7) | 587,513 (42.8) | 7.87 |
| Duration of diabetes, in years, mean (SD) | 9.9 (5.2) | 10.5 (5.2) | 11.67 |
| Diabetes-related complications, n (%) |  |  |  |
| Nephropathy | 3,700 (14.9) | 176,650 (12.9) | 6.03 |
| Retinopathy | 2,395 (9.7) | 130,723 (9.5) | 0.54 |
| Polyneuropathy | 7,214 (29.1) | 340,640 (24.8) | 9.80 |
| Foot syndrome | 5,209 (21.0) | 238,802 (17.4) | 9.29 |
| Comorbidities, n (%) |  |  |  |
| Renal failure | 5,683 (22.9) | 340,589 (24.8) | 4.31 |
| Stroke | 1,486 (6.0) | 101,398 (7.4) | 5.52 |
| Angina pectoris | 1,234 (5.0) | 74,325 (5.4) | 1.92 |
| Myocardial infarction | 1,703 (6.9) | 108,493 (7.9) | 3.89 |
| Acute ischemic heart disease | 202 (0.8) | 12,752 (0.9) | 1.21 |
| Chronic ischemic heart disease | 6,951 (28.1) | 467,278 (34.0) | 12.86 |
| Heart failure | 4,625 (18.7) | 329,903 (24.0) | 13.04 |
| Atherosclerosis of extremities | 2,010 (8.1) | 121,908 (8.9) | 2.71 |
| Peripheral vascular disease | 1,169 (4.7) | 75,861 (5.5) | 3.63 |
| Hypertension | 21,952 (88.6) | 1,216,436 (88.5) | 0.39 |
| COPD | 3,171 (12.8) | 167,204 (12.2) | 1.93 |
| Osteoporosis | 1,091 (4.4) | 86,526 (6.3) | 8.41 |
| Dyslipidemia | 16,308 (65.9) | 888,765 (64.7) | 2.47 |
| Cancer (excluding non-melanoma skin cancer) | 2,871 (11.6) | 200,142 (14.6) | 8.82 |
| Nicotine dependency | 4,384 (17.7) | 182,791 (13.3) | 12.18 |
| Obesity | 15,962 (64.5) | 600,796 (43.7) | 42.54 |
| Depression and anxiety-related disorders | 10,480 (42.3) | 510,728 (37.2) | 10.54 |
| Drug abuse | 317 (1.3) | 13,453 (1.0) | 2.85 |
| Sleep apnoe | 593 (2.4) | 22,188 (1.6) | 5.57 |
| Pancreatitis | 386 (1.6) | 25,222 (1.8) | 2.14 |
| Procedures, n (%) |  |  |  |
| Revascularization | 106 (0.4) | 7,080 (0.5) | 1.27 |
| Coronary bypass/ STENT | 1,022 (4.1) | 60,750 (4.4) | 1.45 |
| Bariatric surgery | 68 (0.3) | 1,986 (0.1) | 2.84 |
| Co-medication, n (%) |  |  |  |
| Benzodiazepines | 622 (2.5) | 34,868 (2.5) | 0.16 |
| Antidepressants | 4,487 (18.1) | 201,345 (14.7) | 9.38 |
| Opioids | 3,003 (12.1) | 154,037 (11.2) | 2.86 |
| Anticonvulsants | 1,829 (7.4) | 88,497 (6.4) | 3.73 |
| ACE inhibitors | 9,654 (39.0) | 539,981 (39.3) | 0.64 |
| Angiotensin II receptor blockers | 10,281 (41.5) | 569,817 (41.5) | 0.10 |
| Beta blockers | 13,599 (54.9) | 800,869 (58.3) | 6.80 |
| Loop diuretics | 5,414 (21.9) | 336,425 (24.5) | 6.21 |
| Other diuretics | 4,927 (19.9) | 287,678 (20.9) | 2.58 |
| Anti thrombotic agents | 7,953 (32.1) | 527,014 (38.4) | 13.08 |
| Calcium channel blockers | 7,513 (30.3) | 416,355 (30.3) | 0.09 |
| Corticosteroids | 142 (0.6) | 8,453 (0.6) | 0.54 |
| Bisphosphonates | 187 (0.8) | 16,399 (1.2) | 4.46 |
| Statins | 13,676 (55.2) | 785,213 (57.1) | 3.86 |
| Fibrates | 420 (1.7) | 18,074 (1.3) | 3.13 |
| Systemic corticosteroids | 2,321 (9.4) | 123,486 (9.0) | 1.34 |
| Glucose-lowering drugs, n (%) |  |  |  |
| Metformin | 20,597 (83.2) | 1,043,856 (76.0) | 17.95 |
| Alpha glucosidase | 71 (0.3) | 4,809 (0.3) | 1.12 |
| Sulfonylureas | 2,339 (9.4) | 113,871 (8.3) | 4.08 |
| DPP-4 inhibitors | 12,302 (49.7) | 578,959 (42.1) | 15.18 |
| Insulin and analogs | 8,585 (34.7) | 372,849 (27.1) | 16.36 |
| Total dispensations of DPP-4 inhibitors and Sulfonylureas, mean (SD) | 1.9 (2.2) | 1.8 (2.3) | 5.90 |
| Total dispensations of insulin and analogs, mean (SD) | 2.1 (4.0) | 1.6 (3.4) | 14.12 |
| Years of continuous SGLT-2 inhibitors treatment, mean (SD) | 1.6 (1.8) | 1.5 (1.7) | 4.20 |
| Health-seeking behaviour, n (%) |  |  |  |
| Influenza vaccination | 9,780 (39.5) | 606,652 (44.1) | 9.45 |
| Breast cancer screening | 727 (2.9) | 31,674 (2.3) | 3.95 |
| Neoplasm screening for men | 3,196 (12.9) | 207,959 (15.1) | 6.42 |
| Colonoscopy | 136 (0.5) | 6,701 (0.5) | 0.86 |
| Neoplasm screening for women | 1,571 (6.3) | 65,653 (4.8) | 6.84 |
| Skin cancer screening | 1,758 (7.1) | 89,765 (6.5) | 2.25 |
| Cohort entry year, n (%) |  |  |  |
| 2013 | 76 (0.3) | 6,597 (0.5) | 2.77 |
| 2014 | 159 (0.6) | 17,398 (1.3) | 6.42 |
| 2015 | 561 (2.3) | 34,042 (2.5) | 1.39 |
| 2016 | 887 (3.6) | 59,021 (4.3) | 3.67 |
| 2017 | 1,238 (5.0) | 85,486 (6.2) | 5.31 |
| 2018 | 1,587 (6.4) | 106,847 (7.8) | 5.33 |
| 2019 | 2,408 (9.7) | 131,173 (9.5) | 0.60 |
| 2020 | 3,147 (12.7) | 155,108 (11.3) | 4.37 |
| 2021 | 4,411 (17.8) | 191,265 (13.9) | 10.67 |
| 2022 | 5,075 (20.5) | 260,069 (18.9) | 3.94 |
| 2023 | 5,216 (21.1) | 327,207 (23.8) | 6.59 |
| Hospital admissions, n (%) |  |  |  |
| 0 | 17,197 (69.4) | 965,835 (70.3) | 1.84 |
| 1 | 4,767 (19.2) | 238,412 (17.3) | 4.92 |
| 2 | 1,680 (6.8) | 95,603 (7.0) | 0.68 |
| ≥ 3 | 1,121 (4.5) | 74,363 (5.4) | 4.07 |
| Outpatient admissions, n (%) |  |  |  |
| ≥ 1 and ≤ 10 | 4,882 (19.7) | 327,747 (23.8) | 10.03 |
| ≥ 11 and ≤ 20 | 13,446 (54.3) | 748,123 (54.4) | 0.29 |
| ≥ 21 and ≤ 30 | 5,214 (21.1) | 247,035 (18.0) | 7.77 |
| > 30 | 1,223 (4.9) | 51,308 (3.7) | 5.92 |
| Federal state, n (%) |  |  |  |
| Unknown | 66 (0.3) | 3,756 (0.3) | 0.13 |
| Schleswig-Holstein | 752 (3.0) | 53,131 (3.9) | 4.55 |
| Hamburg (Hanseatic City) | 260 (1.0) | 16,435 (1.2) | 1.39 |
| Lower Saxony | 1,967 (7.9) | 104,146 (7.6) | 1.36 |
| Bremen (Hanseatic City) | 30 (0.1) | 3,337 (0.2) | 2.86 |
| North Rhine-Westphalia | 6,028 (24.3) | 310,009 (22.6) | 4.21 |
| Hesse | 2,152 (8.7) | 116,152 (8.5) | 0.85 |
| Rhineland-Palatinate | 1,310 (5.3) | 81,275 (5.9) | 2.72 |
| Baden-Württemberg | 1,469 (5.9) | 100,560 (7.3) | 5.57 |
| Bavaria (Free State) | 2,944 (11.9) | 161,257 (11.7) | 0.47 |
| Saarland | 312 (1.3) | 23,607 (1.7) | 3.78 |
| Berlin | 1,040 (4.2) | 57,642 (4.2) | 0.02 |
| Brandenburg | 1,538 (6.2) | 80,809 (5.9) | 1.38 |
| Mecklenburg-Western Pomerania | 1,184 (4.8) | 56,386 (4.1) | 3.29 |
| Saxony (Free State) | 1,313 (5.3) | 81,200 (5.9) | 2.64 |
| Saxony-Anhalt | 1,322 (5.3) | 69,896 (5.1) | 1.13 |
| Thuringia (Free State) | 1,078 (4.4) | 54,615 (4.0) | 1.90 |

ACE: angiotensin-converting enzyme; ASD: absolute standardized difference; COPD: chronic obstructive pulmonary disease; DPP-4: dipeptidyl peptidase 4; GLP-1 RA: glucagon-like peptide-1 receptor agonist; SGLT-2: sodium–glucose cotransporter 2

## **Table S5.** Risk differences for the primary outcome all-cause mortality comparing combination therapy with SGLT-2 inhibitors and GLP-1 RAs versus continued SGLT-2 inhibitor therapy

| **Follow-up time** | **Risk difference (%)** |
| --- | --- |
| 1 year | -0,4% (95% CI -0.7% to -0.1%) |
| 2 years | -0,8% (95% CI -1.3% to -0.3%) |
| 3 years | -1,7% (95% CI -2.3% to -1.1%) |
| 4 years | -1,9% (95% CI -2.8% to -1.1%) |
| 5 years | -2,3% (95% CI -3.7% to -1.0%) |

CI: Confidence interval; GLP-1 RA: Glucagon-like peptide-1 receptor agonists; SGLT-2: Sodium–glucose cotransporter 2

## **Table S6.** Hazard ratios by sex for the primary outcome all-cause mortality comparing combination therapy with SGLT-2 inhibitors and GLP-1 RAs versus continued SGLT-2 inhibitor therapy

| **Subgroup** | **Individuals, n** | **Events** | **Person-years** | **IR^a^ (95%CI)** | **HR^b^ (95%CI)** |
| --- | --- | --- | --- | --- | --- |
| **Men** |  |  |  |  |  |
| Combination therapy  (SGLT-2 inhibitors and GLP-1 RAs) | 11,676 | 270 | 17,264 | 15.6 (13.8–17.6) | 0.75 (0.65–0.87) |
| Continued SGLT-2 inhibitor therapy | 11,712 | 526 | 25,542 | 20.6 (18.9–22.4) | Reference |
| **Women** |  |  |  |  |  |
| Combination therapy  (SGLT-2 inhibitors and GLP-1 RAs) | 9,988 | 140 | 13,396 | 10.5 (8.8–12.3) | 0.64 (0.52–0.78) |
| Continued SGLT-2 inhibitor therapy | 9,952 | 342 | 21,083 | 16.2 (14.5–18.0) | Reference |

^a^ per 1,000 person-years
^b^ Each model was 1:1 time-conditional propensity score matched on hybrid exposure set and nearest neighbour
CI: Confidence interval; HR: Hazard ratio; IR: Incidence rate; GLP-1 RA: Glucagon-like peptide-1 receptor agonist; SGLT-2: Sodium–glucose cotransporter 2;
p-value for interaction by sex: 0.18

## **Table S7.** Hazard ratios by cardiovascular disease status for the primary outcome all-cause mortality comparing combination therapy with SGLT-2 inhibitors and GLP-1 RAs versus continued SGLT-2 inhibitor therapy

| **Subgroup** | **Individuals, n** | **Events** | **Person-years** | **IR ^a^ (95%CI)** | **HR ^b^ (95%CI)** |
| --- | --- | --- | --- | --- | --- |
| **CVD** |  |  |  |  |  |
| Combination therapy  (SGLT-2 inhibitors and GLP-1 RAs) | 9,405 | 293 | 13,028 | 22.5 (20.0–25.2) | 0.68 (0.59–0.78) |
| Continued SGLT-2 inhibitor therapy | 9,236 | 629 | 18,982 | 33.1 (30.6–35.8) | Reference |
| **No CVD** |  |  |  |  |  |
| Combination therapy  (SGLT-2 inhibitors and GLP-1 RAs) | 12,259 | 117 | 17,632 | 6.6 (5.5–8.0) | 0.77 (0.61–0.96) |
| Continued SGLT-2 inhibitor therapy | 12,428 | 239 | 27,643 | 8.6 (7.6–9.8) | Reference |

**^a^** per 1,000 person-years
**^b^** Each model was 1:1 time-conditional propensity score matched on hybrid exposure set and nearest neighbor
CVD was defined as having at least one of the following conditions within one year prior to the study cohort index date: I60x-I64x, I20x, I21x, I22x, I24x, I25x, I739, I702, I50x, I130, I110, I132, 5-361x, 5-362x,5-363x, 8-836x, 8-837x, 8-84.x
CI: Confidence interval; CVD: Cardiovascular disease; HR: Hazard ratio; IR: Incidence rate; GLP-1 RA: Glucagon-like peptide-1 receptor agonist; SGLT-2: Sodium–glucose cotransporter 2;
p-value for interaction by cvd status: 0.34

**REFERENCES**

[1] Suissa S, Moodie EE, Dell'Aniello S. Prevalent new-user cohort designs for comparative drug effect studies by time-conditional propensity scores. Pharmacoepidemiol Drug Saf. 2017; **26**: 459-468

[2] Simms-Williams N, Treves N, Yin H*, et al.* Effect of combination treatment with glucagon-like peptide-1 receptor agonists and sodium-glucose cotransporter-2 inhibitors on incidence of cardiovascular and serious renal events: population based cohort study. BMJ. 2024; **385**: e078242

[3] Lin HD, Lai CL, Dong YH, Tu YK, Chan KA, Suissa S. Re-evaluating Safety and Effectiveness of Dabigatran Versus Warfarin in a Nationwide Data Environment: A Prevalent New-User Design Study. Drugs Real World Outcomes. 2019; **6**: 93-104

[4] Reitzle L, Ihle P, Heidemann C, Paprott R, Koster I, Schmidt C. [Algorithm for the Classification of Type 1 and Type 2 Diabetes Mellitus for the Analysis of Routine Data]. Gesundheitswesen. 2023; **85**: S119-S126

[5] Reitzle L, Schmidt C, Du Y*, et al.* [Estimating prevalent microvascular complications of diabetes mellitus in Germany. Analysis of statutory health insurance data in 2012 and 2013]. Bundesgesundheitsblatt Gesundheitsforschung Gesundheitsschutz. 2020; **63**: 1219-1230

[6] Reitzle L, Koster I, Tuncer O, Schmidt C, Meyer I. [Development and Internal Validation of Case Definitions for Prevalence Estimation of Microvascular Complications of Diabetes in Routine Data]. Gesundheitswesen. 2024; **86**: S196-S204
